# Supplementary material for: Epidemiology, management and outcomes of Cryptococcus gattii infections: A 22-year cohort
Source: PLoS Negl Trop Dis. 2023 Mar 6;17(3):e0011162. doi: 10.1371/journal.pntd.0011162 (PMC10019644; doi:10.1371/journal.pntd.0011162)
Supplement: S2 Table — (PDF) [file pntd.0011162.s002.pdf]

S2 Table: Comorbidities

Comorbidity	Number	Comment
Cigarette smoking	26	4: unknown smoking status
Hazardous alcohol use	16	4: unknown alcohol consumption status
Diabetes mellitus	8	
Chronic kidney disease on haemodialysis	5	
Chronic kidney disease, eGFR<30mL/min not on haemodialysis	4	
Pregnant at time or within 3 months prior to diagnosis	3	
HTLV-1 seropositivity	2	28: no test done
Immunosuppressive medication	2	1: polyarteritis nodosa/ pulmonary vasculitis<br>1: renal transplant
Solid organ malignancy	1	
Immunosuppressive disorder	1	Prior splenectomy
Haematological malignancy	0	
HIV	0	7: no test done

No comorbidities		
No comorbidity evident	3	
Smoking +/- hazardous alcohol intake only	14	
