# Supplementary material for: Epidemiology, management and outcomes of Cryptococcus gattii infections: A 22-year cohort
Source: PLoS Negl Trop Dis. 2023 Mar 6;17(3):e0011162. doi: 10.1371/journal.pntd.0011162 (PMC10019644; doi:10.1371/journal.pntd.0011162)
Supplement: S3 Table — (PDF) [file pntd.0011162.s003.pdf]

Supplementary Table 3: Treatment and outcomes for individuals with fluconazole MIC ≥16 mg/mL and those who received eradication agents other than fluconazole for at least some of their eradication

| Case | Focus | MIC (mg/mL) | Outcome | Surgical resection of cryptococcoma | Fluconazole only used for eradication phase | Duration of induction therapy (days) | Eradication phase |
|---|---|---|---|---|---|---|---|
| 8 | Lung – consolidation 1 lobe | 16 | Cured | No | No eradication phase (non-adherence) | 28 | Prescribed fluconazole but took none |
| 12 | Lung – consolidation 2 lobes | 16 | Cured | No | **Yes** | 14 | Fluconazole 100mg x1.4 years<br>(Dose adjusted for renal function) |
| 18 | CNS and pulmonary - Lung cryptococcoma 9cm | 16 | Persistent infection, improved. | No | No | 56 | Fluconazole x2 days, changed to voriconazole for high fluconazole MIC. Duration indefinite though imaging shows significant improvement after 3 years of voriconazole. |
| 25 | CNS and pulmonary – lung cryptococcoma | 32 | Cured | Yes | No | 42 | Fluconazole 400mg approx. 2 years but also received voriconazole x60 days. Adherence incomplete. |
| 26 | CNS and pulmonary – lung cryptococcoma 8cm | 16 | | Yes | No | 42 | Fluconazole 400mg but developed deranged liver function tests, switched to thrice weekly liposomal amphotericin x1.3 years |

| | | | | | | | |
|---|---|---|---|---|---|---|---|
| 30 | CNS and pulmonary – lung cryptoccoma 1.2cm | 16 | Cured | No | **Yes** | 72 | Fluconazole 400mg x1.2 years |
| 33 | Lung – consolidation 3 lobes | 16 | Cured | No | **Yes** | 23 | Liposomal amphotericin 3/wk for 85 days then fluconazole 200mg x2 years. Died of other cause 3 years later.<br>(Fluconazole dose adjusted for renal function.) |
| 37 | CNS and pulmonary lung cryptococcoma 1.5cm | 16 | Cured with disability | No | **Yes** | 180 | Fluconazole 400mg x1 year |
| 41 | Lung – consolidation 4 lobes | 16 | Cured | No | **Yes** | 42 | Fluconazole x 1.5 years |
| 45 | Lung – cryptococcoma 5.4cm | 16 | Died | No | - | 42 | Developed DRESS reaction while on induction therapy, precipitant uncertain ?beta-lactam. Changed to fluconazole 800mg and transferred interstate. Died three months later, records not available to confirm cause. |
| 47 | Lung – consolidation 4 lobes | 16 | Died | No | - | - | Palliative care initiated while receiving induction treatment, active treatment ceased. |
| Other patients treated with agent other than fluconazole for at least some of their eradication | | | | | | | |

| | | | | | | | |
|---|---|---|---|---|---|---|---|
| 3 | Lung – 2 cryptococcomas, 2.4cm | NR | Cured | No | | 9 | 48 days three times/week conventional amphotericin then fluconazole 400mg planned 5 months |
| 14 | Lung – cryptococcoma 3cm | 8 | Cured | No | | 8 | 23 days fluconazole, developed rash, 81 days itraconazole 200mg. |
| 17 | Lung cryptococcoma 6cm<br>Concurrent CNS infection | 8 | Cured | Yes | | 39 | Changed to voriconazole with concerns for progression on fluconazole however with significant concerns to for treatment adherence. After 13 days, changed back to fluconazole subsequently and completed 18 months eradication, primarily with fluconazole 400mg. |
| 23 | CNS | 4 | Cured | No | | 38 | Developed Severe Cutaneous Adverse Reaction after 27 days fluconazole.<br>Changed to 3/week amphotericin 20 days then itraconazole for a further 3 months. |
| 42 | Lung – approximately 50 cryptoccomas, largest 4cm | 8 | Persistent infection, cure expected | No | | 42 | Received approximately 1 year fluconazole 400mg with concerns for adherence then treatment ceased.<br>12 months later, diagnosed with relapse and commenced on voriconazole. Persistent disease, but significant reduction in size. |

CNS Central nervous system
DRESS drug induced eosinophilia and systemic symptoms
MIC minimum inhibitory concentration
