# Supplementary material for: Epidemiology, management and outcomes of Cryptococcus gattii infections: A 22-year cohort
Source: PLoS Negl Trop Dis. 2023 Mar 6;17(3):e0011162. doi: 10.1371/journal.pntd.0011162 (PMC10019644; doi:10.1371/journal.pntd.0011162)
Supplement: S4 Table — (PDF) [file pntd.0011162.s004.pdf]

S4 Table: Suspected adverse reactions to antifungal drugs requiring change to or withholding of treatment

There were 18 episodes in 14 patients where treatment was changed or withheld due to suspected or confirmed adverse drug events.

One additional person after five weeks of liposomal amphotericin and flucytosine developed a possible Severe Cutaneous Adverse Reaction. This was attributed to a recently commenced anti-bacterial, but an antifungal as the cause could not be excluded. The patient was transferred interstate for higher-level care and died several months later.

Antifungal (Number taking medication at some point of treatment)	Type of adverse reaction	Number of reactions
All		18
Conventional amphotericin (19)		
	AKI	6
	LFT derangement	1
Flucytosine (44)		
	Myelosuppression	3
	LFT derangement	1
Fluconazole (38)		
	LFT derangement	1
	SCAR	1
	Rash	1
Voriconazole (5)		
	LFT derangement	1
	Cytopenia	1
	Unknown	1
Liposomal amphotericin/flucytosine	LFT derangement	1
