# Supplementary material for: Epidemiology, management and outcomes of Cryptococcus gattii infections: A 22-year cohort
Source: PLoS Negl Trop Dis. 2023 Mar 6;17(3):e0011162. doi: 10.1371/journal.pntd.0011162 (PMC10019644; doi:10.1371/journal.pntd.0011162)
Supplement: S5 Table — (PDF) [file pntd.0011162.s005.pdf]

S5 Table: Predictors of death attributed to *Cryptococcus gattii* in a case series of patients with *Cryptococcus gattii* disease; showing final prediction model plus the individual potential predictors excluded from the final model

		Condition Present Effect (Variability)	Condition Absent Effect (Variability)	Comparison[1] IRR	95%CI	P-value
Final multivariate model						
2001 and prior	n (%)	4 of 11 (36.4%)	1 of 34 (2.9%)	89.1	(7.96 to 4,698)	0.0001
Induction ℞ interruption	n (%)	2 of 8 (25.0%)	3 of 37 (8.1%)	62.8	(4.61 to +Inf)	0.0031
No Chronic kidney disease (CKD)	n (%)	2 of 36 (5.6%)	34 of 36 (94.4%)	1.00		
CKD not end-stage	n (%)	1 of 4 (25.0%)	3 of 4 (75.0%)	27.1	(0.69 to +Inf)	0.071
End-stage CKD	n (%)	2 of 5 (40.0%)	3 of 5 (60.0%)	59.7	(4.64 to +Inf)	0.0030
Each line shows the above model plus each excluded covariate estimated separately [2]						
Age at diagnosis	Mean (SD; N)	42.4 (9.6; 5)	39.5 (13.8; 40)	0.67	(0.12 to 3.77)	0.65
Symptom (days) before diagnosis	Mean (SD; N)	21.0 (14.2; 3)	56.0 (85.7; 36)	1.27	(0.60 to 2.70)	0.53
Induction treatment (days)	Mean (SD; N)	56.0 (50.3; 4)	43.8 (29.0; 39)	0.79	(0.48 to 1.32)	0.37
Comorbidities: % of assessed [3]	Mean (SD; N)	0.20 (0.07; 5)	0.16 (0.16; 40)	0.81	(0.48 to 1.37)	0.44
Male	n (%)	3 of 23 (13.0%)	2 of 22 (9.1%)	31.3	(0.14 to 25,337)	0.68
PH DM	n (%)	1 of 8 (12.5%)	4 of 37 (10.8%)	3.40	(0.06 to 61.1)	0.66
PH liver disease	n (%)	1 of 5 (20.0%)	4 of 40 (10.0%)	2.13	(0.03 to 156)	1.00
PH cardiovascular disease	n (%)	1 of 5 (20.0%)	4 of 40 (10.0%)	4.84	(0.12 to +Inf)	0.35
PH connective tissue disease	n (%)	0 of 1 (0.0%)	5 of 44 (11.4%)	0.00	(0.00 to 2.1E+43)	0.63
PH respiratory disease	n (%)	1 of 6 (16.7%)	4 of 39 (10.3%)	1.05	(0.01 to 193)	1.00
Pregnancy at time of diagnosis	n (%)	0 of 3 (0.0%)	5 of 42 (11.9%)	9.8E+14	(0.00 to 2.1E+38)	1.00
PH high alcohol use	n (%)	1 of 16 (6.3%)	4 of 29 (13.8%)	0.84	(0.07 to 17.6)	0.98
Smoking in the last 12 months	n (%)	3 of 26 (11.5%)	2 of 19 (10.5%)	0.84	(0.00 to 289)	1.00
Immune impairment or medicines	n (%)	0 of 3 (0.0%)	5 of 42 (11.9%)	2.57	(0.00 to 244)	1.00
(Immune impairment disease)	n (%)	0 of 1 (0.0%)	5 of 44 (11.4%)	6.60	(0.00 to 628)	1.00
(Immune impairment medication)	n (%)	0 of 2 (0.0%)	5 of 43 (11.6%)	15.0	(0.00 to 657)	1.00
IRIS diagnosis	n (%)	0 of 4 (0.0%)	5 of 41 (12.2%)	6.85	(0.00 to 267)	1.00

Meningitis	n (%)	2 of 27 (7.4%)	3 of 18 (16.7%)	0.34	(0.02 to 4.13)	0.55
Brain cryptococcoma	n (%)	0 of 19 (0.0%)	5 of 26 (19.2%)	1.80	(0.00 to 70.3)	1.00
Lung cryptococcoma	n (%)	4 of 36 (11.1%)	1 of 9 (11.1%)	3.85	(0.15 to 303)	0.67
Blood Culture positive	n (%)	0 of 2 (0.0%)	5 of 43 (11.6%)	4.61	(0.00 to 180)	1.00
Eradication ℞ interruption	n (%)	0 of 6 (0.0%)	5 of 39 (12.8%)	1.62	(0.00 to 63.0)	1.00
CNS surgery	n (%)	2 of 9 (22.2%)	3 of 36 (8.3%)	0.40	(0.02 to 6.10)	0.76
Lung surgery	n (%)	1 of 9 (11.1%)	4 of 36 (11.1%)	0.78	(0.01 to 80.6)	1.00

[1] The effect of various predictors on the rate of all-cause mortality (13 deaths in 45 patients) was estimated as the incidence rate ratio (IRR; 95% confidence intervals; P-values) using exact Poisson regression: the effect of the excluded variables was estimated in 9 separate models of the final model variables plus each other variable: note that an incidence rate ratio of 1.00 indicates no association between that variable and the rate of death; an IRR below 1.00 indicates a negative association, and an IRR above 1.00 indicates a positive association: Missing data were substitute as mean of the whole patient group

[2] Covariates were excluded by backward stepwise regression. The small number of patients mean that false positive and false negative errors are possible, as well as over-specification.
