# Supplementary material for: Epidemiology, management and outcomes of Cryptococcus gattii infections: A 22-year cohort
Source: PLoS Negl Trop Dis. 2023 Mar 6;17(3):e0011162. doi: 10.1371/journal.pntd.0011162 (PMC10019644; doi:10.1371/journal.pntd.0011162)
Supplement: S6 Table — A: Patient and infection characteristics in those with Immune Reconstitution Inflammatory Syndrome (IRIS). B: Neurological disability in those living more than 12 months. (PDF) [file pntd.0011162.s006.pdf]

S6 Table

A: Patient and infection characteristics in those with Immune Reconstitution Inflammatory Syndrome (IRIS)

	IRIS (4)	No IRIS[a] (32)
Male	3 (75%)	16 (50%)
Age <40 years	4 (100%)	11 (34%)
Diagnosed <2013	0	18 (56%)
Diagnosed ≥ 2013	4 (100%)	14 (44%)
Cerebral cryptococcoma	4 (100%)	14 (44%)
CSF opening pressure ≥35cmH_2O or not done due to hydrocephalus	4 (100%)	6/19(32%)
Serum CrAg >1:512	4 (100%)	10/27 (37%)
No co-morbidities, or smoking only	3 (75%)	8/32 (25%)

B: Neurological disability in those living more than 12 months

	Residual neurological disability (4)	No residual neurological symptoms[a] (32)
Brain cryptococcoma	4 (100%)	14 (44%)
Multifocal disease (i.e. lung and CNS)	4 (100%)	13 (41%)
IRIS diagnosis	2 (50%)	2 (6%)

[a]amongst those living more than 12 months
