# Supplementary material for: Epidemiology, management and outcomes of Cryptococcus gattii infections: A 22-year cohort
Source: PLoS Negl Trop Dis. 2023 Mar 6;17(3):e0011162. doi: 10.1371/journal.pntd.0011162 (PMC10019644; doi:10.1371/journal.pntd.0011162)
Supplement: S7 Table — (PDF) [file pntd.0011162.s007.pdf]

S7 Table: Highest opening pressure on lumbar puncture for patients with CNS infection

| | Highest opening pressure | | | | | | | |
|---|---|---|---|---|---|---|---|---|
| | <20 $cmH_2O$ | 20-24 $cmH_2O$ | 25-29 $cmH_2O$ | 30 - 34 $cmH_2O$ | ≥35cm $H_2O$ | Hydrocephalus[a] | Unknown | Total |
| No surgery | 7 | 3 | 2 | 1 | 2 | 2 | 4 | 21 |
| Surgery | 0 | 0 | 0 | 0 | 4 | 2 | 1 | 7 |
| Total | 7 | 3 | 2 | 1 | 6 | 4 | 5 | 28 |

[a]Hydrocephalus on CNS imaging, no lumbar puncture done
