# Supplementary material for: Epidemiology, management and outcomes of Cryptococcus gattii infections: A 22-year cohort
Source: PLoS Negl Trop Dis. 2023 Mar 6;17(3):e0011162. doi: 10.1371/journal.pntd.0011162 (PMC10019644; doi:10.1371/journal.pntd.0011162)
Supplement: Attachment — Submitted filename: OHern_22 years C. gattii_letter response to reviewers.docx [file pntd.0011162.s008.docx]

Dear Dr Joshua Nosanchuk, MD,

Thank you for giving us the opportunity to submit a revised draft of our manuscript titled “Epidemiology, management and outcomes of *Cryptococcus gattii* infections: A 22 year cohort” to *PLOS Neglected Tropical Diseases.* We appreciate the time and effort that you and the reviewers have dedicated to providing feedback and are grateful for your insightful comments. We have incorporated most of the suggestions.

Pleases see below for a point-by-point response to the reviewers’ comments and concerns in blue. In the manuscript we have highlighted changes in blue.

Sincerely

Jennifer O’Hern

Reviewer's Responses to Questions


Reviewer #1: The objective, study design and population are clearly stated and appear appropriate.
In the abstract, it is stated as “retrospective case series” and in the main text, it is stated “retrospective cohort” study. It seems to be a combination of both.

Abstract wording has been changed to retrospective cohort study for consistency.

Authors provide a comprehensive, longitudinal description of the epidemiology, management, and outcomes of Cg infection. The authors explored factors associated with mortality, immune reconstitution inflammatory syndrome, neurologic sequelae and persistence or relapse. There are no concerns for ethical or regulatory issue.

Additional comments:
*Lines 118: It is not clearly stated what the comparison groups were in the univariable analysis.

The univariable analysis was excluded from the final draft so this line has been deleted.

*Lines 89; 376. Authors discuss cure and relapse. A limitation to consider is that the authors may have under-estimated relapse due to insufficient follow up time, especially for the cases diagnosed during the latter period.

Discussion line 552 – added:

“Relapse may be underestimated in those diagnosed later in the study.”

*Lines 84-88:
Authors discuss IRIS in this section.
It is not entirely clear if IRIS is the correct terminology (as there was no clear “immune reconstitution”, although that could be debatable) or if PIIRS would be the more appropriate terminology.  However, there does not appear to be clear consensus on this terminology in general (not referring to this study) and PIIRS terminology was only introduced in 2015. Authors do reference a study on PIIRS (ref 25, line 445) in the discussion section and appear to be interpreting IRIS and PIIRS interchangeably. While this point may not be completely relevant to this study, could be a limitation in accurate classification of IRIS.

Thank you for highlighting these different descriptors and apologies for the erroneous impression that PIIRS and IRIS are interchangeable. Our understanding is that Williamson((1) proposed PIIRS in 2015 as: ‘a new syndrome of cryptococcal post-infectious inflammatory response syndrome (PIIRS) defined in previously healthy patients with cryptococcal meningo-encephalitis as the presence of a poor clinical response in the setting of at least 1 month of amphotericin-based fungicidal therapy and sterile cerebrospinal cultures.’

By contrast, IRIS is commonly understood to be a flare of inflammation due to immune recovery occurring after initial improvement and conversion of cultures to negative. Practitioners in our setting use the term IRIS and this seems widely understood. The proposed mechanism in HIV negative individuals has been described by Einseidel et al, doi.org/10.1086/424746: the cryptococcal capsule directly inhibits phagocytosis and may induce a state of relative immunosuppression through the effects of such components as glucuronoxylomannan. Thus, immune restoration can follow successful initial treatment, leading to a vigorous inflammatory response and worsening of symptoms.

Hence we prefer to use IRIS instead of PIIRS, but now provide an explanation of the hypothesised mechanism for IRIS in HIV-negative individuals as follows:

We have added below comments to the discussion:

Lines 535 onwards:

“The mechanism of IRIS in HIV negative individuals with *C. gattii* is incompletely understood(2). IRIS is hypothesized to occur in this setting when the transient immunosuppression or immune-evasion, attributed to direct effects of cryptococcal capsule components, resolves with successful treatment, leading to an exaggerated inflammatory response and worsening of clinical and/or radiological findings(3-5)”

Reviewer #2: See below

--------------------

Results


Reviewer #1: Overall, the analysis match the analysis plan and the results are clearly and completely presented. Few comments below:

*Table 5:
The small number of events limit the number of variables that can be included in the model. The wide CI likely reflects the high variability of the estimates due to the small sample size. It may help the readers to clarify which variables were included as adjustment variables and consider including few pre-specified variables (that are biologically relevant or confounders) given limited number of events and small sample size. Readers will need to interpret the findings with the limitations in mind.

We acknowledge the limitations of our analysis due to small sample size. We note in the discussion results should be interpreted carefully and regarded as exploratory. We think however it is important to incorporate the multivariable analysis model to explore confounding factors that may affect outcomes.

We have included excluded variables in the Supplementary Data (Supplementary Table 4):

Supplementary Table 4: Predictors of death attributed to *Cryptococcus gattii* in a case series of patients with *Cryptococcus gattii* disease; showing final prediction model plus the individual potential predictors excluded from the final model

*Line 134: “All except one were Aboriginal Australians, predominantly from the Arnhem regions of the Northern Territory (Figure 2)..”
-Authors may have meant to state, “Table 1” instead of “Figure 2” here.

Amended to Table 1 and Figure 1 (Note figure 1 depicts the regions from which people came.)

*Table 1.
The denominator number is not always included. May be better to be consistent. Could include denominator in all cells. Alternatively, can remove the denominator number but include a total n in the top row and indicate in the footnote if there are few missing.

Denominator data now included.

*Line 184, authors state “All received induction therapy with systemic amphotericin..”
Was that the case in patients with pulmonary only disease as well?

Yes. This is a very important issue and we have now clarified this, incorporating a more comprehensive response to Reviewer 2 point 7 (Management) to explain the rationale for longer treatments used in our setting.

Line 498 onwards:

“Patients in our study generally received prolonged induction therapy with an amphotericin formulation (median 6 weeks, minimum 8 days, Table 4). There is no sound evidence base to inform the safe minimum treatment duration for different forms of *C. gattii* infection. *C. gattii* is recognized to be more complicated to treat than *C. neoformans*(6-8). Australian guidelines from 2013 recommend 2 weeks induction therapy for isolated pulmonary diseases (8) whereas our regional guidelines (NT) previously recommended four weeks minimum induction treatment for all forms of *C. gattii* disease (unpublished local guidelines). This approach contrasts with IDSA guidance and other expert advice support shorter intravenous (induction) courses for both *C. neoformans* and *C. gattii* pulmonary infections in those at lower risk of failure(7, 9) or even use of oral azole therapy only for isolated pulmonary infection. Recent trial findings of success of single high-dose amphotericin and 5-flucytosine induction for *C. neoformans* meningitis in HIV-positive people (10) raise consideration of whether shorter intravenous courses may also be possible for *C. gattii*. In our setting, this needs to be balanced against the challenges of ensuring oral medication supply, adherence and patient follow-up in people returning to very remote areas of Northern Australia. Our data however did show that cure was achievable if <14 days induction therapy was given in pulmonary disease (though numbers were very small, n=2) and cure was also achievable with <28 days of intravenous induction therapy in all forms of disease but with a relapse rate of 1/9 (11%). Relapse also occurred in one individual despite 6 weeks of intravenous therapy. We conclude that regional guidelines can be revised to recommend that intravenous induction therapy duration for *C. gattii* infection be tailored to burden of disease, comorbidities and ability to access follow-up out of hospital care, from two weeks for uncomplicated pulmonary disease to prolonged (potentially months) for extensive, persistently culture positive disease. “

*Line 235: Authors state “Death was not associated with age, anatomical site of infection…”
However, the data on association between anatomical site of infection and death is not presented in table 5.

Please see Table 5 and also Supplementary Table 4 – adjusted to include this information.

*Table 1
It may help the readers if the authors give a clearer definition of how “CNS disease only” and “pulmonary disease only” were categorized. It may be of interest given the higher mortality in pulmonary disease compared to CNS disease (as presented in table 4).

For example, in table 1, two patients with “pulmonary disease only” had CNS symptoms. Would it be correct to assume that the patients had a lumbar puncture and CSF cultures were negative ruling out CNS disease despite the symptoms?

This was based on clinical impression however 14/16 patients with “pulmonary disease” only underwent lumbar puncture (mostly also with CNS imaging) and 7/8 with CNS infection had negative pulmonary imaging at time of diagnosis. This information has been added to the results, line 142 to 144:

“Multifocal disease was most common (20) followed by pulmonary disease alone (16) (with negative lumbar puncture with or without imaging in 14/16), central nervous system (CNS) disease alone (8) (with negative pulmonary imaging confirmed in 7/8) and fungaemia without identifiable focus (1).”

The two patients referred to in Table 1 with pulmonary disease only with CNS symptoms had negative lumbar punctures and CNS imaging. This note has been added as a footnote to Table 1.:

^c^ lumbar puncture and CNS imaging (CT or MRI) both normal

*In Table 1, authors may consider including the variables they found to be associated with mortality (e.g., ESKD, treatment interruption)

We have included both ESKD and treatment interruption in Table 1.

*May be helpful to clarify how “treatment interruption” was defined.

Definition has been added – see line 86-88

“Interruption to induction treatment was defined as no anti-cryptococcal antifungal therapy for more than 96 hours/4 days after initial commencement due to a patient-initiated treatment break (usually premature self-discharge from care) or medically-planned withhold (usually for adverse effects).”

Reviewer #2: See below

--------------------

Conclusions

Reviewer #1: *It is interesting that disease with Cg was mostly seen in Australian Aboriginal who were relatively young, median age of 41, majority with comorbid conditions. What may be some of the reasons this group of patient population are particularly vulnerable? Or is it more reflective of the general patient population in Northern Australia? Would it be possible other socioeconomic factors may be in play? How generalizable are the findings to Cg cases in other parts of the world?

The Northern Territory has a relatively young median age and this is a population with a generally high burden of disease associated with social determinants of health. A large proportion of the patients in this study live remotely and spend a large amount of time outdoors. Discussion regarding this has been added to the discussion - see lines 417-431:

“The cohort in this study were relatively young compared to other studies(11-13), likely related to the marked low median age in this region (32 for Northern Territory in the 2016 census)(14). As seen in other studies for *C. gattii*, unlike *C. neoformans,* many patients lacked clinical evidence of overt immunosuppression (11, 13, 15). Recently it has been suggested that *C. gattii* infection may be promoted by defects in the immune system not previously recognized with recent studies suggesting an association with Anti-GM-CSF autoantibodies in some patients with *C. gattii* infection.(16, 17) This has yet to be assessed in our cohort.

Knowledge gaps in the environmental and host factors underpinning local disease epidemiology remain unanswered. Every adult in this case series was of Aboriginal ethnicity; the only non-Aboriginal person was a child from a neighbouring Asian country evacuated to Australia for management. To what extent this disparity reflects environmental exposure risk versus host risk factors is unclear. Specific acquired or intrinsic immune pertubations not demonstrable on current routine laboratory may be of importance; but the more relevant issue is to address the burden of chronic conditions related to social determinants of health, which may be playing a role in increasing host susceptibility to infection.”

.

*May consider elaborating on why smoking and alcohol use were included as comorbid conditions, their relevance with Cg infection.

Smoking has been found to be associated with *C. gattii* in several studies (12, 13, 15).

Some studies find high alcohol intake is associated with *C. gattii (13)* and hazardous alcohol use is a known risk factor for other endemic environmental infections in northern Australia including *Acinetobacter* (18) and melioidosis (19). This was therefore included given its association with immunosuppression and also possibly poorer health outcomes and social factors that may delay health care consultation. However, in the interests of paper length, we have not included these explanations in the paper.

*The treatment delay from time of symptom onset is notable. What were the factors that led to the delay? Was it because clinicians did not suspect the disease during the earlier period, or patients present with atypical symptoms or healthcare access issue? Are there ways improvements can be made?

The time from hospital presentation to diagnosis was actually usually quite short (median 3 days). We were not able to collect primary care data to determine duration and frequency of primary care contacts prior to evacuation from remote settings to hospital and therefore are unable to answer to what extent pre-hospital diagnosis delay was due to patient factors (delayed health seeking) versus clinician factors (not suspecting or testing for cryptococcal infection).

The median duration of time from presentation to diagnosis has been included in the results to clarify this.

Line 179: “Time to diagnosis and treatment from presentation to hospital was a median of 3 day [IQR 1,9].”

*The decrease in mortality in the latter period is notable. Could it be that more milder cases were detected in the latter period with increased or improved use of diagnostics?

We believe the decrease in mortality is more likely attributable to improved treatment, since disease extent did not appreciably change over time. We are not aware of improved uptake of diagnostics: Cryptococcal antigen has been widely used for about 15 years and the increase in case numbers applied to culture positive cases also. We have added in line 523-524:

“We speculate that medical, surgical and intensive care approaches have improved over time accounting for the lower later morality.”

*It is interesting that pulmonary cryptococcomas is associated with higher morbidity or mortality compared to CNS disease and that symptom duration before presentation was shorter in pulmonary vs CNS disease, which seem to differ from that due to Cn infection in HIV. As the authors present, optimal management/treatment approach between Cg and Cn infection likely differ and need to be studied more. This study would be an important contribution to the existing literature.

Thank you.

Note, although mortality was highest in those with pulmonary disease, the multivariable analysis found that mortality was associated with ESKD and treatment interruption, which occurred more commonly in the goup with pulmonary disease. We prefer not to read too much into the symptom duration, since this information is subject ot recall and documentation biases.

Reviewer #2: See below

--------------------

<b>Editorial and Data Presentation Modifications?</b></br><br/>
Use  this section for editorial suggestions as well as relatively minor modifications of existing data that would enhance clarity. If the only modifications needed are minor and/or editorial, you may wish to recommend “Minor Revision” or “Accept”.

Reviewer #1: Minor revision

Reviewer #2: The manuscript (in particular the Results and Discussion sections) is very long and detailed. I would suggest making the report more concise, and moving some parts of the data to the Appendix.

The information from subsection in results “Adverse Drug Reactions” (paragraph starting ~line 229) has been presented as a Table in the Supplementary data. Please see other new edits throughout to reduce the word length.

--------------------

Summary and General Comments

Reviewer #1: Authors present an extensive case description of a relatively rare disease over 22 years and make insightful and novel points. The comparison of mortality between early vs. late period is interesting and may reflect changing epidemiology, therapeutics, and/or learning curve with management. The finding of rise in fluconazole MIC over time may have important public health implication and perhaps use of voriconazole or other newer therapeutics need to be studied more.

This study will make an important addition to the existing body of the literature. The paper also uncovers several important questions that we have little data in with limited guidelines (e.g., monitoring of azole MIC and use of fluc vs vori; surgical vs medical management of cryptococcomas; diagnosis/management of IRIS). The study emphasize the need for better diagnostics and therapeutics.

Authors report important limitations of their study including single center study and small sample size that limited powered analysis. There was likely residual confounding or confounding by indication when examining outcomes by surgical vs medical approach as the authors discuss.

Thank you for this feedback.

Reviewer #2: O’Hern and colleagues performed a single center retrospective study looking at the epidemiology, management and outcomes of Cryptococcus gattii infections managed at Royal Darwin Hospital, Northern Territory, Australia. Forty-five cases were included, diagnosed over a 22-year study period (Jan 1996 to Sept 2018). The main study on this question is the Australia-wide retrospective study done by S. Chen and colleagues, in which 86 patients from 17 institutions were included (study period: 2000 – 2007). The study done by O’Hern and colleagues is interesting because their local setting is very particular, with First Nations Australians in this region having a particularly high incidence of C. gattii infection, and specific characteristics.

Here are my comments and suggestions:

1_ The manuscript (in particular the Results and Discussion sections) is very long and detailed. I would suggest making the report more concise, and moving some parts of the data to the Appendix.

2_ Definitions used:
-       How was the ‘date of diagnosis’ defined?

We have added this definition:

Lines 72-73

“Date of diagnosis was defined as the date the first test result confirming diagnosis was reported and the treating team’s impression was of Cryptococcal infection.

-       The authors state that the geographical location of likely exposure was determined from patient’s main residence ‘in the prior 12 months’. Do they refer to the 12 months before diagnosis, or before onset of symptoms? This is important because the time from onset of symptoms to diagnosis is sometimes very long in patients with cryptococcosis.

We have clarified this:

Lines 68-70

“The geographical location of likely exposure was determined from patient’s main residence in the prior twelve months prior to symptom onset, or prior to diagnosis if patient asymptomatic.”

-       An elevated CSF opening pressure was defined as ≥ 35 cmH20. Please justify the choice of this high threshold.

Our apologies for creating the impression that only CSF pressure ≥ 35 cmH_2_0 was considered elevated – this was not the intention. We consider any pressure >20 cmH_2_0 to be elevated, but in the methods we described that very high readings e.g. beyond the end of the manometer, were assigned a value of 35 cmH_2_0 to allow for numerical calculations. The methods state (line 89-92):

“CSF opening pressure was recorded as 35 cmH_2_0 for the purpose of statistical calculations if it was documented as ‘>34 cmH_2_0’ or ‘high’.”

Line 90-91: We have added ‘where ‘high’ indicated pressure to or beyond the top of the manometer’. Also we have clarified in line 339 that pressure >35 was ‘very’ elevated.

3_ Flow chart: a significant proportion of the patients (n=12) were excluded because they were ‘not managed at study hospital’ (Royal Darwin Hospital). Were these patients diagnosed at Royal Darwin Hospital? Systematically excluding this important proportion of the eligible patients may be associated with a high risk of bias, given that these patients possibly had significantly different characteristics, managements, and outcomes.

These patients were diagnosed at a hospital outside the Top End region (i.e. in Central Australia). This hospital refers some microbiological specimens to the Royal Darwin Hospital. These patients are less likely to live in the Top End Region and were excluded as were not managed at the Royal Darwin Hospital.

This has been clarified with a comment on the flowchart:

“Note: 12 not managed at study hospital – these are cases diagnosed at another hospital and managed exclusively outside the Top End region. They were identified as the other hospital refers some microbiological specimens to the study hospital laboratory for processing.”

4_ Comorbidities: Many patients had few or no major comorbidities. Please discuss that recent studies suggested that C. gattii infections may be promoted by relatively subtle immunocompromising conditions that are not routinely searched for, such as the presence anti-GM-CSF antibodies (Kuo et al, CID 2021)

This has been added to the discussion.

Line 417 onwards (as already noted above):

“The cohort in this study were relatively young compared to other studies(11-13), likely related to the marked low median age in this region (32 for Northern Territory in the 2016 census)(14). As seen in other studies for *C. gattii*, unlike *C. neoformans,* many patients lacked clinical evidence of overt immunosuppression (11, 13, 15). Recently it has been suggested that *C. gattii* infection may be promoted by defects in the immune system not previously recognized with recent studies suggesting an association with Anti-GM-CSF autoantibodies in some patients with *C. gattii* infection.(16, 17) This has yet to be assessed in our cohort.”

5_ Incidence of cryptococcosis (Table 2): the local incidence of C. gattii infection has greatly increased in the last period (2015-2018). Could this be explained by a change in diagnostic methods? e.g., was the CrAg latex test replaced by the more sensitive and now widely used LFA?

Our data do not provide answers for the apparent increase in case numbers: whether due to population increases, improved uptake of testing, improved sensitivity of tests, or true increase due to increased human incursion into existing environmental niches or expanded environmental niches. LFA was introduced after 2018 and therefore does not explain higher detection in the 2015-2018 period. Because of the length of the manuscript and the request to limit length, we have not added this speculation to the paper but would be happy to if the editor wishes.

.

6_ Cryptococcus CNS involvement may be asymptomatic. It appears that most patients with CNS involvement presented with neurological symptoms (see Table 1). Please state whether a lumbar puncture (± brain imaging) was systematically done at time of diagnosis of cryptococcosis, even in the absence of neurological symptoms (e.g., in patients presenting with signs of C. gattii lung infection only)

Lumbar puncture and usually CNS imaging is a routine part of work-up for those diagnosed with pulmonary cryptococcosis. Similarly chest imaging is routine in those diagnosed with CNS cryptococcosis. There were very few patients that did not get this. We have summarised this by adding to the results.

Line 141-144:

“Multifocal disease was most common (20) followed by pulmonary disease alone (16) (with negative lumbar puncture in 14/16, negative CNS imaging 12/16), central nervous system (CNS) disease alone (8) (with negative pulmonary imaging confirmed in 7/8) and fungaemia without identifiable focus (1)”

7_ Management:
a)      Patients with C. gattii infection received prolonged induction therapy (i.e., amphotericin-based therapy), with generally at least 6 weeks given for CNS disease, and a median of 3 weeks given for lung disease only (Table 4). This is in line with Australian guidelines for the treatment of cryptococcosis (S. Chen et al, IMJ 2014), but the evidence supporting this recommendation seems to be of particularly low quality. Please comment on this important point. Are there any comparative data on this question? What are the potential advantages and disadvantages of prolonged induction therapy? Given that the recently published AMBITION RCT has shown that C. neoformans meningitis can be treated with single-dose liposomal amphotericin B (combined with flucytosine and fluconazole), could amphotericin-based therapy be shortened in selected patients with C. gattii infection? (e.g., patients with meningitis but no cryptococcoma, or patients with lung infection only)

Please see response to reviewer 1 on this matter. We have added to the discussion to consider this:

Line 498 onwards:

“Patients in our study generally received prolonged induction therapy with an amphotericin formulation (median 6 weeks, minimum 8 days, Table 4). There is no sound evidence base to inform the safe minimum treatment duration for different forms of *C. gattii* infection. *C. gattii* is recognized to be more complicated to treat than *C. neoformans*(6-8). Australian guidelines from 2013 recommend 2 weeks induction therapy for isolated pulmonary diseases (8) whereas our regional guidelines (NT) previously recommended four weeks minimum induction treatment for all forms of *C. gattii* disease (unpublished local guidelines). This approach contrasts with IDSA guidance and other expert advice support shorter intravenous (induction) courses for both *C. neoformans* and *C. gattii* pulmonary infections in those at lower risk of failure(7, 9) or even use of oral azole therapy only for isolated pulmonary infection. Recent trial findings of success of single high-dose amphotericin and 5-flucytosine induction for *C. neoformans* meningitis in HIV-positive people (10) raise consideration of whether shorter intravenous courses may also be possible for *C. gattii*. In our setting, this needs to be balanced against the challenges of ensuring oral medication supply, adherence and patient follow-up in people returning to very remote areas of Northern Australia. Our data however did show that cure was achievable if <14 days induction therapy was given in pulmonary disease (though numbers were very small, n=2) and cure was also achievable with <28 days of intravenous induction therapy in all forms of disease but with a relapse rate of 1/9 (11%). Relapse also occurred in one individual despite 6 weeks of intravenous therapy. We conclude that regional guidelines can be revised to recommend that intravenous induction therapy duration for *C. gattii* infection be tailored to burden of disease, comorbidities and ability to access follow-up out of hospital care, from two weeks for uncomplicated pulmonary disease to prolonged (potentially months) for extensive, persistently culture positive disease.”

b)      Were antifungal susceptibility testing results reported to treating physicians? If yes, how did that influence antifungal management?

Yes, physicians had access to susceptibility results, and used these to guide therapeutic choices – mentioned in line 221 and 310 and Supplementary Table 3.

We have also added in the discussion line 482-485:

“We did observe clinician preference to use voriconazole later in the study period for *C. gattii* with fluconazole ≥16 μg/mL, though its use was often limited by toxicity and in such patients there was usually a good outcome when treatment subsequently reverted to fluconazole.”

.

8_ Outcomes: the authors performed a multivariable analysis to identify factors associated with mortality in patients with C. gattii infection.
-       Please clearly define what outcome variable was used for this analysis. Was it all-cause mortality, or death attributed to C. gattii? Please also confirm that this was at 12 months post-diagnosis of cryptococcosis, not at last follow-up visit.

The outcome variable was death due to *C. gattii,* not all cause mortality. This is noted in the text )see Results - Outcomes).

(Note cause of death is defined in methods – by review of death certificate and patient notes – see line 105).

-       The relevance of doing a multivariable analysis appears limited. First, the number of deaths was very low (5 related deaths/45 patients), and prediction models using small data sets lead to very uncertain predictions, as illustrated by the very wide 95% CIs obtained. Second, the clinical relevance of performing a multivariable analysis is limited by the observations that only one death occurred among cases diagnosed after 2002, and that most deaths occurred in patients with lung infection only.

As noted above, we acknowledge the limitations of our analysis due to small sample size. We note in the discussion results should be interpreted carefully and regarded as exploratory. We think however it is important to incorporate the multivariable analysis model to explore confounding factors that may affect outcomes.

-       The variable ‘induction treatment interruption’ was selected as an independent variable, for this multivariable analysis (this was the only management variable used). I would recommend not using this type of variable given the retrospective design of the study, and the high risk of bias associated with these variables.

Whilst acknowledging this concern, the variable is carefully defined as >4 days (definition added to methods) and we think it remains important to include. There were 8 patients who had one or more significant interruptions to their treatment and this was a significant factor in treatment and outcome. Because we have made the interruption >4 days and have carefully reviewed notes and charts for this, we think we have minimised risk of bias.

1.	Williamson PR. Post-infectious inflammatory response syndrome (PIIRS): Dissociation of T-cell-macrophage signaling in previously healthy individuals with cryptococcal fungal meningoencephalitis. Macrophage (Houst). 2015;2.

2.	Chen SC, Meyer W, Sorrell TC. Cryptococcus gattii infections. Clin Microbiol Rev. 2014;27(4):980-1024.

3.	Beardsley J, Sorrell TC, Chen SC. Central Nervous System Cryptococcal Infections in Non-HIV Infected Patients. J Fungi (Basel). 2019;5(3).

4.	Einsiedel L, Gordon DL, Dyer JR. Paradoxical inflammatory reaction during treatment of Cryptococcus neoformans var. gattii meningitis in an HIV-seronegative woman. Clin Infect Dis. 2004;39(8):e78-82.

5.	Beardsley JD, A; Keighley, C; Garnham, K; Halliday, C; Chen, SC; Sorrell, TC. What’s new in *Cryptococcus gattii*: From bench to bedside and beyond. Journal of Fungi. 2023;9(1).

6.	Howard-Jones AR, Sparks R, Pham D, Halliday C, Beardsley J, Chen SC. Pulmonary Cryptococcosis. J Fungi (Basel). 2022;8(11).

7.	Perfect JR, Dismukes WE, Dromer F, Goldman DL, Graybill JR, Hamill RJ, et al. Clinical practice guidelines for the management of cryptococcal disease: 2010 update by the infectious diseases society of america. Clin Infect Dis. 2010;50(3):291-322.

8.	Chen SC, Korman TM, Slavin MA, Marriott D, Byth K, Bak N, et al. Antifungal therapy and management of complications of cryptococcosis due to Cryptococcus gattii. Clin Infect Dis. 2013;57(4):543-51.

9.	Fisher JF, Valencia-Rey PA, Davis WB. Pulmonary Cryptococcosis in the Immunocompetent Patient-Many Questions, Some Answers. Open Forum Infect Dis. 2016;3(3):ofw167.

10.	Jarvis JN, Lawrence DS, Meya DB, Kagimu E, Kasibante J, Mpoza E, et al. Single-Dose Liposomal Amphotericin B Treatment for Cryptococcal Meningitis. N Engl J Med. 2022;386(12):1109-20.

11.	Galanis E, Macdougall L, Kidd S, Morshed M, British Columbia Cryptococcus gattii Working G. Epidemiology of Cryptococcus gattii, British Columbia, Canada, 1999-2007. Emerg Infect Dis. 2010;16(2):251-7.

12.	Phillips P, Galanis E, MacDougall L, Chong MY, Balshaw R, Cook VJ, et al. Longitudinal clinical findings and outcome among patients with Cryptococcus gattii infection in British Columbia. Clin Infect Dis. 2015;60(9):1368-76.

13.	Hughes CM, Lennon D, Davis JS. CRyptOcoccosis in Newcastle and the hUnTer (CRONUT) - An epidemiological study. Infect Dis Health. 2020;25(1):34-42.

14.	Statistics ABo. Snapshot of Northern Territory www.abs.gov.au/articles/snapshot-nt-2021: Australian Bureau of Statistics; 2022 [updated 28/6/2022.

15.	Chen SC, Slavin MA, Heath CH, Playford EG, Byth K, Marriott D, et al. Clinical manifestations of Cryptococcus gattii infection: determinants of neurological sequelae and death. Clin Infect Dis. 2012;55(6):789-98.

16.	Kuo CY, Wang SY, Shih HP, Tu KH, Huang WC, Ding JY, et al. Disseminated Cryptococcosis Due to Anti-Granulocyte-Macrophage Colony-Stimulating Factor Autoantibodies in the Absence of Pulmonary Alveolar Proteinosis. J Clin Immunol. 2017;37(2):143-52.

17.	Rosen LB, Freeman AF, Yang LM, Jutivorakool K, Olivier KN, Angkasekwinai N, et al. Anti-GM-CSF autoantibodies in patients with cryptococcal meningitis. J Immunol. 2013;190(8):3959-66.

18.	Meumann EM, Anstey NM, Currie BJ, Piera KA, Kenyon JJ, Hall RM, et al. Genomic epidemiology of severe community-onset Acinetobacter baumannii infection. Microb Genom. 2019;5(3).

19.	Currie BJ, Jacups SP, Cheng AC, Fisher DA, Anstey NM, Huffam SE, et al. Melioidosis epidemiology and risk factors from a prospective whole-population study in northern Australia. Trop Med Int Health. 2004;9(11):1167-74.
